# Risks Associated with 5α-Reductase Inhibitor Use: Analysis of Adverse Drug Reactions Reported to EudraVigilance

**DOI:** 10.3390/ph19060939

**Published:** 2026-06-15

**Authors:** Ricardo Alves, Samuel Silvestre, Cristina Monteiro

**Affiliations:** 1 Faculty of Health Sciences, University of Beira Interior, 6200-506 Covilhã, Portugal; ricardopmalves@gmail.com; 2 RISE-Health, Department of Chemistry, Faculty of Sciences, University of Beira Interior, 6201-001 Covilhã, Portugal; sms@ubi.pt; 3 RISE-Health, Department of Medical Sciences, Faculty of Health Sciences, University of Beira Interior, 6200-506 Covilhã, Portugal; 4 UFBI-Pharmacovigilance Unit of Beira Interior, Faculty of Health Sciences, University of Beira Interior, 6200-506 Covilhã, Portugal

**Keywords:** 5α-reductase, dutasteride, finasteride, benign prostatic hyperplasia, androgenic alopecia, pharmacovigilance, adverse drug reaction, EudraVigilance

## Abstract

**Background/Objectives**: 5α-Reductase inhibitors (5ARIs) are commonly used to treat and prevent androgenic alopecia and benign prostatic hyperplasia. Despite their well-established effectiveness, they are associated with adverse drug reactions (ADRs), highlighting the need for continuous safety assessment. This study aimed to analyze the ADRs associated with finasteride and dutasteride, both as monotherapy and in combination therapy. **Methods**: A retrospective analysis of ADRs associated with finasteride and dutasteride reported to EudraVigilance between 1 January 2005 and 27 March 2023 was performed. A total of 7777 reports were selected, and various variables were examined, including the temporal evolution of ADR reports, reporter profile, and the age group of the affected population. ADRs were categorized based on their seriousness and outcome, with particular focus on the most common reactions and their alignment with the Summary of Product Characteristics (SmPC). **Results**: The most affected age group, excluding the “Not Specified” category, was “18–64 years.” Overall, finasteride was the most reported. The majority of reported ADRs were classified as “Serious,” with a predominant outcome of “Persists without recovery,” and a significant proportion of these ADRs were not listed in the respective 5ARI SmPCs. Among the ADRs classified as “Serious,” the most frequently reported seriousness criterion was “Clinically important”. **Conclusions**: The results emphasize how crucial it is to continuously monitor these drugs in order to prevent and mitigate ADRs, ensure population safety, and promote public health. Additionally, more research is required to determine whether the ADRs not included in the SmPC could be new warning signs.

## 1. Introduction

According to the World Health Organization, pharmacovigilance is defined as “the science and activities related to the detection, assessment, understanding, and prevention of adverse effects or any other drug/vaccine-related problems.” [1]. The development of a drug is a highly complex process, consisting of several phases and has a prolonged duration, which can extend over a period of 10 to 15 years. Although pre-marketing clinical trials provide important information regarding the efficacy and safety of medicines, several adverse drug reactions, particularly rare, long-term, or population-specific events, may only become evident during the post-marketing phase through pharmacovigilance activities and real-world evidence generation. In fact, only through the use of the drug in real-world conditions can a more comprehensive understanding of its risks and benefits be obtained. Pharmacovigilance is an essential tool for detecting, preventing, and minimizing adverse drug reactions (ADRs). This practice ensures the protection of patients and the safeguarding of public health, ensuring that marketed drugs maintain their safety and therapeutic efficacy throughout their entire life cycle and not just during the research and development phase [2,3,4].

In Europe, the European Medicines Agency (EMA) is responsible for coordinating the scientific resources supplied by Member States for the assessment, oversight, and monitoring of drug safety. The EMA coordinates pharmacovigilance activities through the EudraVigilance system. This system is a data processing and management platform intended for the reporting and evaluation of suspected adverse reactions, applicable both during the drug development stages and following their marketing approval within the European Economic Area [5,6]. These real-world pharmacovigilance data are essential for identifying potential safety signals and supporting continuous benefit–risk assessment throughout the lifecycle of medicines.

**Figure 1:**
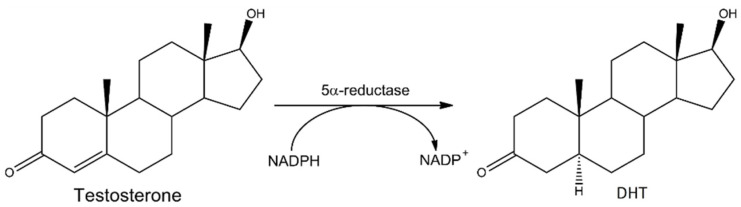
Conversion of testosterone into 5α-dihydrotestosterone (DHT) catalyzed by the 5α-eductase enzyme, in the presence of the cofactor (NADPH).

5α-Reductase is an intracellular enzyme that converts androgen testosterone into dihydrotestosterone (DHT) (Figure 1) [7]. Among the different isoforms identified, type I and type II are the most clinically relevant. DHT plays an important physiological role in males; in fact, an abnormal DHT activity has been associated with androgen-dependent disorders, particularly benign prostatic hyperplasia (BPH) and androgenetic alopecia (AGA) [8,9]. BPH is characterized by the progressive enlargement of the prostate due to increased proliferation of prostatic cells, leading to lower urinary tract symptoms and reduced quality of life. This condition is strongly associated with aging and, although it rarely poses a life-threatening risk, it can significantly impact the patient’s quality of life. The increase in life expectancy has made BPH a relevant public health issue, particularly due to its growing impact on the elderly population. Treatment is indicated only for those whose BPH causes symptoms that affect their quality of life. However, there are exceptions to this rule when BPH results in serious complications, even in the absence of significant symptoms. BPH can progress asymptomatically, causing severe and sometimes irreversible changes to the bladder, kidneys, and other organs. This highlights the importance of regular assessments and monitoring to prevent the silent progression of the disease and avoid severe complications [10]. AGA is a hereditary pattern of hair loss characterized by the conversion of terminal hair into miniaturized vellus hairs [9]. This condition is highly prevalent, affecting up to 80% of Caucasian men and 50% of women by the age of 70. However, the patterns of hair loss change according to gender. Hair loss associated with AGA can cause emotional distress and negatively impact the quality of life of affected individuals, often leading them to seek treatment. Finasteride and dutasteride are commonly used to stabilize hair loss and promote hair growth, demonstrating efficacy in managing this condition [9].

Since DHT plays a central role in the pathophysiology of both conditions, reducing DHT production has become an important therapeutic strategy [8,9,11].

5α-Reductase inhibitors (5ARIs), including finasteride and dutasteride, are important in treating conditions like BPH and AGA by lowering DHT levels, a crucial androgen in the development of both conditions [12]. Finasteride functions as a competitive inhibitor of the intracellular enzyme 5α-reductase type II [7]. The United States Food and Drug Administration (FDA) initially approved it in 1992 [12]. It is a widely prescribed drug for the treatment of BPH at a daily dose of 5 mg and for the treatment of AGA at a daily dose of 1 mg. This drug reduces DHT levels, which are responsible for the proliferation of prostatic cells and the miniaturization of hair follicles associated with AGA [8]. Finasteride has been shown to suppress serum DHT levels by approximately 70%. Its oral bioavailability is about 80% and is not affected by food intake. Maximum plasma concentrations are reached approximately two hours after administration, with complete absorption occurring between six and eight hours later [12].

Dutasteride, the first dual inhibitor of 5α-reductase type I and type II isoenzymes, was approved by the FDA in 2001. It is primarily used for the treatment of BPH at a daily dose of 0.5 mg. Due to its broader ability to inhibit 5α-reductase and, consequently, DHT production, dutasteride has also been used, although unofficially, in the treatment of AGA, with emerging evidence of its efficacy in reducing hair loss associated with this condition [13]. In patients with BPH treated with 0.5 mg/day of dutasteride, an average reduction of 94% in serum DHT levels was observed after one year and 93% after two years. The time required to reach peak serum concentration after the oral administration of a single 0.5 mg dose of dutasteride ranges from 1 to 3 h. Its absolute bioavailability is approximately 60% and, like finasteride, is not affected by the presence of food [14]. Both drugs in this therapeutic class demonstrate efficacy in reducing DHT levels and, consequently, in treating the aforementioned conditions. However, the differences in selectivity and the extent of 5α-reductase inhibition may influence the choice of treatment, depending on the specific clinical condition and the individual patient’s response to the medication [7,8,13].

Both finasteride and dutasteride are considered well-tolerated drugs, with most reported ADRs being mild and transient. The adverse effects most frequently associated with these drugs are related to sexual dysfunctions. The increase in reports of sexual-related ADRs in post-marketing led many countries to amend the Summary of Product Characteristics (SmPC) to include some of these ADRs. Several other adverse effects have been described, including depressive states and cardiovascular problems [7,9,14]. In recent years, increasing attention has also been given to the potential neuropsychiatric adverse effects associated with 5α-reductase inhibitors, particularly finasteride. Several studies and pharmacovigilance investigations have reported associations with depression, anxiety, emotional disturbances, and suicidal ideation, especially among younger patients treated for androgenetic alopecia. These concerns have gained growing recognition within the scientific community and among regulatory agencies, including the EMA, the FDA, and the Medicines and Healthcare products Regulatory Agency, leading to strengthened safety warnings and increased regulatory monitoring regarding psychiatric adverse reactions associated with finasteride use [15].

This study’s main goal was to identify, compile, organize, and evaluate reports of ADRs associated with 5α-reductase inhibitors, identified as suspected drugs, used by adult men and reported to the EudraVigilance database, between 1 January 2005 and 27 March 2023. Through this analysis, we aim to contribute to improving patient safety by identifying and understanding potential ADRs associated with the use of these 5ARIs.

## 2. Results

### 2.1. Qualification of Reports over the Years

**Figure 2:**
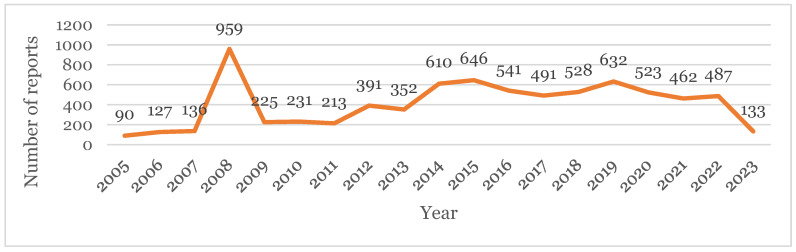
Reports registered annually between 2005 and 2023.

The analysis of Figure 2 reveals an overall upward trend in reports over time till 2019. A gradual increase is observed up until 2015, with a notable peak in 2008, which recorded 959 reports (the highest value of the entire analyzed period), followed by a decrease in 2009. After this year, the number of reports fluctuated over time without a consistent linear trend.

### 2.2. Characterization of Reports According to the Type of Reporter

Of the total 7777 reports sent to EudraVigilance, 2480 were submitted by non-healthcare professionals (patients/consumers or caregivers), accounting for 31.89% of the overall reports. In contrast, 5297 reports were made by healthcare professionals, representing 68.11% of the total.

### 2.3. Characterization of the Population by Age Group

**Figure 3:**
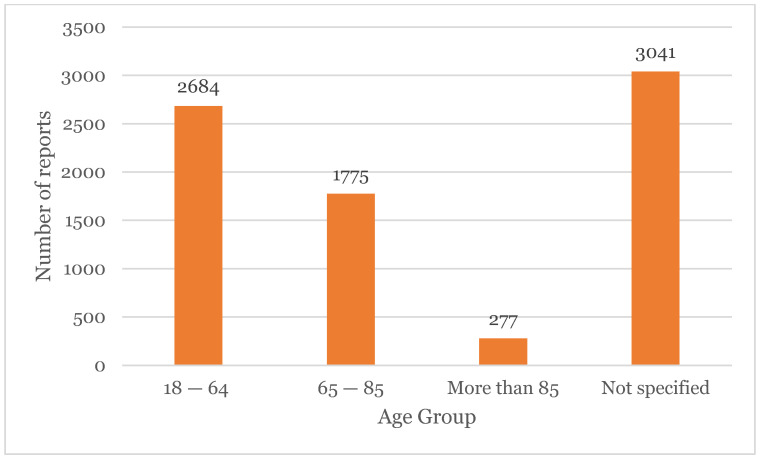
Characterization of the population according to age group.

To characterize ADRs based on age group, the population was divided into four categories: 18–64 years, 65–85 years, over 85 years, and unspecified. The largest proportion corresponds to the “Not Specified” category, with 3041 reports, representing 39.10% of the total. The age group of 18 to 64 years follows with 2684 reports, accounting for 34.51%. On the other hand, the age group over 85 years presents the lowest number of cases, with 277 reports, representing 3.56% of the 7777 reports analyzed (Figure 3). Finasteride-related reports were predominantly associated with individuals aged 18–64 years (41.65%) and with cases in which the age was not specified (41.25%). In contrast, dutasteride-related reports were more frequently associated with individuals aged 65–85 years (43.04%). Reports involving the combination of both 5ARIs represented only a small proportion of cases across all age groups (Table 1).

### 2.4. Quantification of Reports by 5α-Reductase Inhibitor

The reports were analyzed individually, including cases where dutasteride and finasteride were considered as isolated suspected drugs, as well as cases where the combination of both was considered the suspected cause of the reported ADRs. Finasteride stood out as the most frequently suspected drug, being associated with 5614 reports, representing 72.19% of the total cases. In second place, dutasteride was associated with 2135 reports, corresponding to 27.45% of the total. Lastly, the combination of both drugs was identified as the suspected cause in only 28 reports, which accounts for 0.36% of all recorded reports.

### 2.5. Characterization of Reported Adverse Drug Reactions

#### 2.5.1. Distribution of Adverse Reactions by Seriousness and by Seriousness Criteria

Of the 7777 reports reported, 2153 were classified as “Non-serious,” representing 27.68% of the total. In contrast, reports categorized as “Serious” totaled 5624 cases, corresponding to 72.32%. The serious adverse reaction reports were further categorized according to five criteria of seriousness: “Clinically Important,” “Disability,” “Hospitalization,” “Life-Threatening,” and “Death.” More than half (2854 reports, or 50.75%) were assigned to the “Clinically Important” criterion. Next, 1176 reports (20.91%) were classified as “Hospitalization.” The “Disability” criterion was applied to 896 cases, representing 15.93% of the serious adverse reactions. The “Death” and “Life-Threatening” classifications were assigned to 409 (7.27%) and 289 (5.14%) cases, respectively.

#### 2.5.2. Characterization of the Clinical Status of the Patients Regarding the Evolution of Adverse Reactions

Among the 7777 reports analyzed, the evolution “Persists without recovery” was the most frequent, assigned to 2705 cases, representing 34.78% of the total. The “Unknown” criterion was the second most frequent, with 2364 reports (30.40%). The third most common evolution was “Recovery,” applied to 1358 cases, corresponding to 17.46% of the total reported. The “In recovery” criterion was recorded in 853 reports (10.97%). The evolution with a fatal outcome, “Death,” occurred in 409 cases (5.26%). Lastly, the least assigned evolution was “Recovery with sequelae,” with only 88 cases, representing 1.13% of the total reported.

#### 2.5.3. Relationship Between Seriousness and 5α-Reductase Inhibitor and Age Group

**Figure 4:**
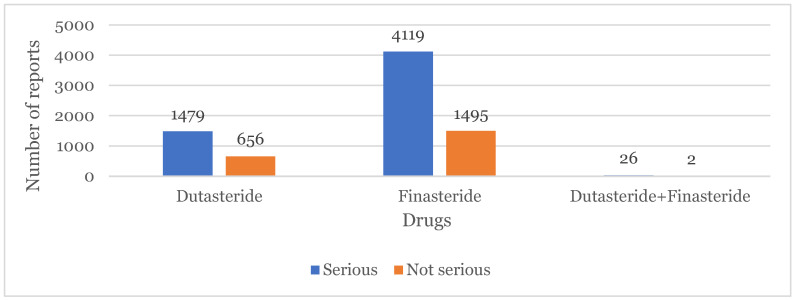
Relationship between the seriousness and suspected 5α-reductase inhibitor.

The analysis (Figure 4) began with dutasteride, identified as the suspected drug in 2135 reports. Of these, 1479 (69.27%) were classified as “Serious,” while the remaining 656 (30.73%) were categorized as “Non-serious.” Regarding finasteride, of a total of 5614 reports in which this drug was suspected, 4119 were classified as “Serious” (73.37%). The remaining 1495 cases were categorized as “Non-serious” (26.63%). For the reports where the combination of dutasteride and finasteride was considered the suspected cause of the ADRs, 28 reports were analyzed. Of these, 26 were classified as “Serious” (92.86%), while the remaining 2 were categorized as “Non-serious” (7.14%). A statistically significant association was observed between the suspected 5ARI and the seriousness classification of ADR reports (χ^2^ = 50.58; *p* < 0.001).

From the observation of Figure 4, it is clear that for all three contexts analyzed (dutasteride and finasteride used individually, as well as their combination), the majority of ADR reports were classified as “Serious.” However, the combination of both drugs showed a larger discrepancy between the seriousness categories, with the highest proportion of serious cases (92.86%) compared to non-serious cases (7.14%).

**Figure 5:**
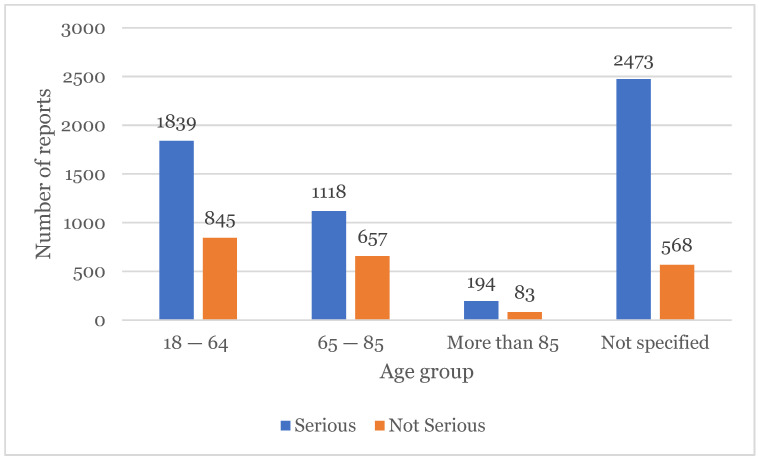
Relationship between the seriousness and age group associated with adverse reactions.

Regarding the relationship between the seriousness of reported ADRs and the age group of affected patients, as previously referred, the analyzed age groups were 18 to 64 years, 65 to 85 years, over 85 years, and “Unspecified.” It was observed that, across all age groups, the number of ADRs classified as “Serious” exceeded the number of ADRs classified as “Non-serious” (Figure 5). In the 18-to-64 years age group, 1839 ADRs were classified as “Serious” (68.52%) and 845 as “Non-serious” (31.48%). In the 65-to-85 years age group, 1118 ADRs were classified as “Serious” (62.99%), while 657 were considered “Non-serious” (37.01%). In the age group above 85 years, which had the fewest reported cases, 194 ADRs were classified as “Serious” (70.04%) and 83 as “Non-serious” (29.96%). In the “Unspecified” age group, 2473 ADRs were classified as “Serious” (81.32%) and 568 as “Non-serious” (18.68%), which is the highest ratio between “Serious” and “Non-serious” ADRs. A statistically significant association was observed between age group and the seriousness classification of ADR reports (χ^2^ = 196.62; *p* < 0.001). The highest proportion of reports classified as serious was observed in the “Not specified” age group (81.32%).

#### 2.5.4. The Distribution of Adverse Reactions Described in the Summary of Product Characteristics

In total, 38,448 ADRs were analyzed. Of these, only 10,687 ADRs (approximately 27.80%) were described in the SmPCs, while 27,761 ADRs (72.20%) were not listed in the SmPCs, representing about three-quarters of all the ADRs analyzed.

When analyzing the 5α-reductase inhibitors individually, in the case of dutasteride, the majority of reported ADRs were not described in the SmPC. Out of a total of 4803 reported ADRs, only 697 (14.51%) were referenced in the SmPCs. The remaining 4106 ADRs, which represent 85.49% of the total, were not described in the SmPCs. Regarding finasteride, of the 33,553 reported and analyzed ADRs where finasteride was considered the primary suspected cause, only 9975 (29.73%) were described in the SmPCs. In contrast, the vast majority, amounting to 23,578 ADRs (70.27%), were not mentioned in the SmPCs. Of the 92 ADRs reported and analyzed in which the combination of dutasteride and finasteride was the suspected cause, only 15 (16.30%) were described in the SmPCs. In contrast, the majority, representing 77 ADRs (83.70%), were not listed in the SmPCs.

Table 2 displays the eight most frequently reported ADRs associated with the 5α-reductase inhibitors, along with their frequency as described in the SmPCs. The most frequently reported ADR was “Erectile dysfunction,” also known as “Sexual impotence,” with a total of 1961 reports. This was followed by “Decreased libido,” which accounted for 1427 reports.

Table 3 provides the four most frequently reported ADRs not included in the SmPC. The most commonly reported ADR was “Cognitive impairment,” with 623 reports. This was followed by “Fatigue,” with 529 reports, and “Insomnia,” which accounted for 377 reports.

#### 2.5.5. Characterization of the Most Prevalent Adverse Reactions with Terms Belonging to the Designated Medical Event List

Of all the ADRs analyzed in this study, 266 ADRs whose terms are included in the Designated Medical Event (DME) list were identified. Dutasteride was suspected for 97 ADRs included in the DME list, while finasteride was associated with 169 ADRs. However, the combination of both 5ARIs did not present any ADRs distributed among the terms in the DME list (Table 4). In total, the 266 identified ADRs were distributed across 27 terms from the DME list. Regarding the description in the SmPC, only the term “Angioedema” was included, while all other ADRs were not described in their respective SmPCs.

## 3. Discussion

The conducted study allowed for the characterization of reported cases and the ADRs associated with the use of 5ARIs, specifically dutasteride and finasteride, both in monotherapy and in combination. The data were obtained from the EudraVigilance database, covering the period from 1 January 2005 to 27 March 2023.

Regarding the number of reports received over time, despite some fluctuations, there was a general trend of growth in the number of suspected ADR reports, particularly until 2019. This increase may be directly related to the population growth in Europe, as well as the rise in life expectancy [16]. The fact that the European population is increasingly reaching older ages, which are more vulnerable to the occurrence of ADRs, coupled with the growing trend of medication use with advancing age, makes it plausible that the combination of these factors has contributed to the increase in ADRs and, consequently, to the rise in reported ADRs [17,18,19]. Another possible explanation could lie in the implementation of regulations in 2012 that reformed European pharmacovigilance legislation. These guidelines enabled non-healthcare professionals to also report suspected ADRs in EudraVigilance [20,21]. Educational and informational campaigns directed at both healthcare professionals and the general population, covering a range of topics from the promotion of rational drug use to guidelines on how to report ADRs correctly and effectively, may also have contributed to the increase in reports [22,23,24,25]. In 2008, a significant peak was observed in the number of ADR reports, making it the year with the highest number of reports among all the analyzed years. A possible explanation for this increase could be related to the fact that, in the same year, the Swedish Medicines Agency conducted a safety investigation and issued a warning indicating that the use of finasteride could cause irreversible sexual dysfunction [26]. As a result, the Agency required the manufacturer to update the product leaflet, including difficulty in achieving an erection as a potential side effect, which could persist indefinitely. These changes in the finasteride leaflets may have raised greater awareness among both healthcare professionals and users, leading to an increase in reports.

Since 2015, there was stagnation in the growth of reports, with a slight decrease observed until 2017. However, in 2017, the number of reports began to rise again, maintaining this upward trend until 2019. It is plausible that this increase in reports in 2017 was triggered by the development of new features in the EudraVigilance database. Additionally, in 2017, several German pharmaceutical companies began submitting their reports exclusively to the European EudraVigilance database, further contributing to this increase in reports [27]. After 2019, a decrease in the number of reports was observed until the end of the analyzed period. One of the factors that may have contributed to this reduction was the continuous evolution of the EudraVigilance database, which, over the years, has developed its data review and analysis methods. Reports began to be examined more thoroughly and rigorously, leading to the exclusion of many cases considered invalid, such as duplicate reports, from the database, which would not have been detected as invalid in the past [28]. Some other explanations can account for the underreporting observed after 2019, such as the belief that only serious ADRs should be reported, the misconception that medications available on the market are entirely safe, based on the argument that if they were not, they would have already been withdrawn, the lack of feedback from national regulatory authorities in response to submitted forms, procrastination in sending ADR reports, and a lack of time or motivation to complete the reports [29,30]. Another explanation for the decrease in ADR reports of 5ARIs after 2019 could be the COVID-19 pandemic which began in late 2019/early 2020. With vaccination campaigns taking place in the following years and limited time for the preclinical and clinical studies required for the development of the vaccines, there was an increased concern, both from users and healthcare professionals, regarding the monitoring of ADRs associated with these recent vaccines. In contrast, this situation led to less attention being given to ADRs from all other medications, including 5ARIs [31].

Regarding the type of reporter, ADRs can be reported by healthcare professionals or non-healthcare professionals. Although this study started in 2005 and non-healthcare professionals were only formally categorized as reporters after the legislative change in 2012, they still represented 31.89% of the total reports analyzed [32,33]. This growing contribution from non-healthcare professionals is important as it, in collaboration with healthcare professionals, helps to build a more comprehensive and detailed database on the safety profiles of medications, thereby strengthening and enhancing pharmacovigilance systems [34]. However, it was still the healthcare professionals who contributed the most to the reporting of suspected ADRs in this study. The contribution of healthcare professionals is especially crucial because, with their specialized scientific knowledge, they are able to report in greater detail, providing information and technical specifics about the ADRs, thus contributing higher quality data to pharmacovigilance [35].

About the age groups, the categories “Not Specified” and “18–64” contributed the most to the reports. This age range, which includes individuals between 18 and 64 years old, represents the largest portion of the European population, which is consistent with the data recorded in this study [36]. It is relevant to consider, as a possible justification for the fact that the “18–64” age group contributed the most to the reports, that this group has the largest range, covering individuals of 46 different ages, while the other age groups encompass fewer distinct ages. This broader age range in the “18–64” group may naturally have influenced a higher proportion of ADR reports. A possible explanation for the fact that the “Not Specified” age group represented a significant portion of the population affected by ADRs (39.10%) could be related to the minimum requirements needed for submitting reports to the EudraVigilance database. For a report to be valid, only one piece of identifying information about the patient needs to be provided, such as initials, birthdate, age, age range, or sex. Since these minimal criteria are sufficient, it is possible that some reports were made without specifying the age range, resulting in a higher proportion of reports in this age group [37].

Regarding the 5ARI suspected of causing ADRs, most of the cases studied (72.19%) involved finasteride. This higher number of reports involving finasteride may be related to the fact that, in this study, no distinction was made between the 1 mg and 5 mg dosages, resulting in all reports related to finasteride being grouped together as a single category, thus leading to a high number of ADRs associated with this drug. This approach ultimately covered a broader population compared to dutasteride, as finasteride is indicated, at a dosage of 1 mg, for the treatment of AGA and, at a dosage of 5 mg, for the treatment and management of BPH and the prevention of urological events [38]. On the other hand, dutasteride is predominantly used only for the treatment of moderate to severe symptoms of BPH. However, some studies have shown that, due to its greater potency in inhibiting the conversion of testosterone to DHT, dutasteride may still demonstrate superior efficacy to finasteride, even in the treatment of AGA [11,39,40]. The higher number of reports involving finasteride as the suspected drug causing ADRs, considering that dutasteride received its marketing authorization more recently, contradicts the theory that people are generally more vigilant and cautious about ADRs associated with medications introduced more recently to the market, reporting them more frequently [31]. It is also important to highlight that a possible justification for the high number of reports associated with finasteride is the fact that this drug is prescribed more frequently than dutasteride by doctors, as evidenced by a population-based study published in BMC Musculoskeletal Disorders [38]. Only 28 cases reported ADRs associated with the simultaneous use of both 5ARI inhibitors, which is consistent with the scarcity of evidence and studies supporting the combination of these drugs, except for a study conducted with low doses of the combination for the treatment of AGA [41]. It is also possible that these ADRs attributed to the combination of the 5ARIs may have resulted from cases of experimental self-medication or irrational use of the drugs. As reported in some studies, there is no evidence to support that the combination of the two 5ARIs is more effective than the isolated use of dutasteride or finasteride. Furthermore, combining medications without proper medical guidance can increase the risk of adverse effects due to drug interactions or the excessive use of substances with similar mechanisms of action [41].

When classifying the ADRs in terms of seriousness, it was found that the “Serious” category predominated over the “Non-serious” category. One possible explanation for this predominance may be the misconception that only serious ADRs are important, leading to a greater perceived need for their reporting [29,42]. Another possible explanation for the higher number of serious reports could be that healthcare professionals are more sensitized to report serious ADRs, as these are typically associated with higher healthcare costs and increased morbidity [43]. However, this result does not align with studies that indicate that 5ARIs are generally well-tolerated in terms of adverse effects [9]. Subsequently, the serious reports were characterized according to seriousness criteria, with the most prevalent category being “Clinically important.”

The progression of “Serious” ADRs were analyzed to determine their implications on patients’ lives and to more effectively evaluate the benefit–risk balance of 5ARIs. Based on the results obtained, it was found that, in most cases, the progression was classified as “Persists without recovery,” followed by the categories “Unknown” and “Recovery.” Some previously conducted research studies mention the association of finasteride use with persistent sexual and psychological adverse effects. Considering that many of the ADRs analyzed were of a sexual and psychological/psychiatric nature, this association may explain the high rate of cases classified as “Persists without recovery” [44,45].

Finasteride, as the suspected drug associated with ADRs, showed a high rate of serious reports compared to non-serious ones, with 73.37% of reports classified as serious. In contrast, dutasteride had 69.27% of its reports classified as serious. Considering the proximity of the serious and non-serious report ratios for both drugs (69.27% and 73.37%, respectively), we can state that these data align with some studies suggesting that both finasteride and dutasteride have similar rates of ADRs [11]. The discrepancy observed in the evaluation of “Serious” ADR reports compared to “Non-serious” in finasteride may also be associated with reports of persistent ADRs that lasted at least three months after discontinuing finasteride, as observed in the last decade. These persistent ADRs gave rise to the term “post-finasteride syndrome,” suggesting that finasteride may be associated with more serious ADRs compared to dutasteride [46]. However, there is still no solid scientific basis determining the prevalence and incidence of persistent ADRs associated with finasteride, and post-finasteride syndrome is not fully recognized by the scientific community [46].

When analyzing the relationship between the seriousness of ADRs and age group, excluding the “Unspecified” age group, it was found that the greatest discrepancy between ADRs classified as “Serious” and “Non-serious” occurred in the age group corresponding to individuals over 85 years. However, it is important to note that in this age group, the number of reported cases is significantly lower compared to the other age groups. A plausible explanation for the higher incidence of serious ADRs in this older age group (over 85 years) is that the risk of ADRs is age-dependent, meaning that it increases due to the pharmacokinetic and pharmacodynamic changes in metabolism associated with aging, as well as polypharmacy, which is more common among the elderly [11,47]. Subsequently, the reported ADRs were compared with the information described in the SmPCs of the 5ARIs. The majority of the identified ADRs were not listed in the SPCs. Among the numerous reported ADRs that are not described in the SmPCs, those such as “Cognitive disorder,” “Fatigue,” “Insomnia,” and “Hypertension” were most frequently observed. The ADR “Cognitive disorder” has been mentioned in some bibliographic studies that establish a correlation between lower levels of DHT and cognitive decline, both in animal models and in human studies [48]. The ADR “Insomnia” has also been addressed in some studies, which suggest that the type 2 isoform of 5α-reductase is highly expressed in the thalamic reticular nucleus in adult male rats. This anatomical region is crucial for sleep regulation, and the inhibition of this isoform may be associated with insomnia induction [48]. The ADR “Chronic fatigue” has been discussed in several studies, particularly in the context of ADRs associated with post-finasteride syndrome [46]. A hypothesis for the occurrence of this ADR suggests that 5α-reductase inhibition may interfere with the metabolism of glucocorticoids and mineralocorticoids, affecting their clearance. These metabolic alterations could have a direct impact on the body’s energy balance and response to physiological stress, which would explain the onset of chronic fatigue in some patients [46,49]. The ADR “Hypertension” is not typically discussed as a side effect directly associated with 5ARIs. However, a possible explanation for the significant impact of this ADR observed in the study could be related to the fact that 5ARIs are associated with a slight increase in anxiety, a condition that, in turn, may trigger or worsen hypertension. Additionally, it is important to consider that 5ARIs are frequently prescribed to older individuals, in whom the risk of hypertension is naturally higher due to the aging process [50]. Another plausible explanation for the hypertension observed in many cases could stem from the fact that the inhibition of the 5α-reductase enzyme interferes with the metabolism of mineralocorticoids, as mentioned earlier [46]. Aldosterone is the most important mineralocorticoid, playing a crucial role in regulating sodium and water homeostasis in the body. The excess aldosterone caused by the inhibition of 5α-reductase, in combination with a high salt load, can lead to episodes of hypertension and potentially cause cardiac damage [51]. The eight most reported ADRs described in the SmPCs showed three different frequencies: “Frequent,” “Uncommon,” and “Unknown”. Among them, “Erectile dysfunction” and “Decreased libido” stood out as the most reported and described in the SmPC. These ADRs are also widely reported in several studies, which support the frequency attributed to them, indicating that they are already recognized as ADRs strongly associated with the profile of 5ARIs [44,48]. The third and fourth most reported ADRs described in the SmPCs, “Depressive symptoms” and “Anxiety,” respectively, are classified with frequencies as “Unknown.” Although there are several references to these ADRs in various articles, research on them is limited, and the mechanisms of action that could explain these ADRs remain, at this time, unclear. This lack of clarity in the mechanisms may hinder the understanding of the relationship between 5ARIs and these ADRs, highlighting the need for further studies to clarify these issues [44,52]. However, a possible explanation for the manifestation of depressive symptoms and anxiety could be associated with alterations in the metabolism of glucocorticoids caused by the inhibition of 5α-reductase, which may lead to higher levels of cortisol in the body [46]. Under normal circumstances, when the body faces stressful situations, the hypothalamic–pituitary–adrenal axis is activated, leading to the release of glucocorticoids such as cortisol. Elevated cortisol levels are associated with chronic stress states, which can contribute to the development of depressive and anxiety symptoms [53]. The fifth most described ADR in the SmPC, “Sexual dysfunction,” was reported 960 times. However, this ADR has a broad definition, which ultimately encompasses other ADRs related to sexual performance that were also reported and analyzed throughout this study, complicating the specificity and accuracy of the data analysis [54]. However, in general, the results observed are generally consistent with previously published pharmacovigilance analyses involving 5α-reductase inhibitors from other international databases, including the FDA Adverse Event Reporting System and World Health Organization VigiBase. Previous studies similarly identified sexual dysfunction, psychiatric adverse reactions, gynecomastia, and persistent adverse effects among the most frequently reported safety concerns associated with finasteride and dutasteride [55,56,57]. In particular, Nguyen et al. reported a significant disproportionality signal for suicidality, depression, and anxiety associated with finasteride use, especially among younger patients treated for androgenetic alopecia [55]. Likewise, investigations based on VigiBase and FAERS data also identified persistent sexual dysfunction and psychiatric ADRs as important emerging safety signals related to 5ARIs [56]. These observations are generally aligned with the ADR patterns identified in the present EudraVigilance-based analysis. However, the differences observed in ADR patterns across studies may partially reflect the heterogeneity of the treated populations, since finasteride and dutasteride are prescribed for distinct clinical conditions involving different age groups, clinical characteristics, psychological impacts, comorbidities, and concomitant therapies. These factors may contribute to variability in the frequency and nature of reported adverse drug reactions, particularly regarding neuropsychiatric outcomes. Nevertheless, differences in reporting frequencies and ADR distribution between databases may occur due to variations in regional prescribing patterns, population characteristics, regulatory awareness, and spontaneous reporting practices. Despite these differences, the overall consistency between the present findings and previously published pharmacovigilance data reinforces the importance of continuous post-marketing surveillance of finasteride and dutasteride [9].

Regarding the terms included in the DME list, a total of 266 ADRs associated with the studied 5ARIs were analyzed, resulting in the identification of 27 distinct terms. Among these, the term “Deafness” stands out, accounting for 43 ADRs. There is a scarcity of specific studies directly linking deafness to the administration of finasteride or dutasteride. Following these are the terms “Angioedema” and “Pancreatitis”. It is important to highlight that, among these terms, only “Angioedema” is described in the SmPCs of both dutasteride and finasteride (12,14). The presence of several DME terms not described highlights the potential emergence of new safety signals. To ensure this safety, the EMA conducts continuous reviews of medications in circulation. In the specific case of 5ARIs, the EMA recently expressed concerns regarding ADRs, including suicidal behaviors and ideation. In this regard, on 4 October 2024, a safety review was initiated for medications containing finasteride and dutasteride, aiming to investigate these ADRs, as outlined in the Information Circular, issued by the EMA [57].

This study has several limitations related to the reported ADRs. A major limitation is the lack of specificity in ADR reporting. In many cases, broad terms such as “Sexual dysfunction” were used, while in others more specific terms (e.g., “Erectile dysfunction”) were reported. This heterogeneity complicates data analysis and interpretation. Additionally, some reports also contained incomplete or ambiguous information on ADRs, limiting the robustness of the analysis, partly due to the restricted level of detail available in publicly accessible data. Another important limitation is the absence of drug utilization data, which prevents the estimation of incidence rates and limits the ability to contextualize the frequency of reported ADRs. Furthermore, due to the intrinsic limitations of spontaneous reporting pharmacovigilance databases, more advanced risk quantification methods could not be reliably applied. Nevertheless, despite these limitations, spontaneous reporting systems such as EudraVigilance remain essential tools in pharmacovigilance, as they allow the identification of potential safety signals, rare adverse drug reactions, and clinically relevant patterns that may contribute to the continuous monitoring of medicine safety and support future regulatory assessments. Another important limitation of this study is the fact that there was no distinction between ADR reports associated with finasteride 1 mg and 5 mg due to the lack of detailed and consistently available dosage information in the publicly accessible EudraVigilance database. Both dosages were therefore analyzed together to maximize the number of eligible reports and provide a broader overview of finasteride-associated ADRs. However, it is important to acknowledge that these dosages are commonly prescribed for different clinical indications, namely, androgenetic alopecia and benign prostatic hyperplasia, involving populations with potentially different demographic characteristics, comorbidities, co-medications, and ADR profiles.

## 4. Materials and Methods

A retrospective analysis of spontaneous reports available in the EudraVigilance database, which is publicly accessible through the website https://www.adrreports.eu/pt/index.html (accessed on 21 April 2024) [58], was performed. The study focused on reported cases of medications within the 5ARI therapeutic class, specifically dutasteride and finasteride. While the study was primarily centered on these two drugs, the reports were examined considering dutasteride, finasteride, and their combination. ADRs were assessed independently for each suspected drug when either dutasteride or finasteride was identified as the principal cause. Furthermore, ADR reports involving both drugs in combination were also analyzed, even though the combined use of these medications is not commonly prescribed [41]. No distinction was made between ADR reports associated with the use of finasteride at different dosages (1 mg and 5 mg) to maximize the amount of available data for analysis and, consequently, to obtain a broader and more representative sample of ADRs, regardless of the administered dose. However, it is important to acknowledge that these dosages are commonly prescribed for different clinical conditions, namely, AGA and BPH, involving populations with potentially different demographic characteristics, comorbidities, co-medications, and ADR profiles. The data analyzed covered the period from 1 January 2005 to 27 March 2023. The date of March 27 corresponds to the point at which data collection from the EudraVigilance database was conducted. Statistical analysis was performed using Microsoft Office Excel 365, Version 2605 (Microsoft Corporation, Redmond, WA, USA), which facilitated the organization of the data according to the variables under study. Subsequently, appropriate tables and charts were created to aid in the interpretation and visualization of the results obtained, with the aim of providing a clear and structured analysis of the ADR reports associated with 5ARIs. Quantitative variables were expressed as absolute frequencies and percentages. Associations between categorical variables were assessed using the chi-square (χ^2^) test, considering *p* < 0.05 as statistically significant, for specific dates.

This study considered several variables to characterize the reports related to 5ARIs. Among the analyzed variables, the type of reporter, differentiating between healthcare professionals and non-professionals (patients or caregivers), and the demographic profile of the population, with a particular focus on the age group of individuals, were analyzed. Regarding the analysis of ADRs, both total reports and those attributed to each suspected 5ARI were quantified. ADRs were also classified based on different parameters, including seriousness, seriousness criteria, and the clinical progression of the ADRs. Additionally, a detailed analysis was conducted on the presence or absence of ADRs in the SmPCs of the suspected drugs, enabling an evaluation of the alignment between the ADRs reported and the information officially described in the SmPCs. Reports were also characterized based on terms included in the DME list. The DME list is a compilation created by the EMA, primarily aimed at identifying ADR reports involving events considered rare and serious [59]. It is important to note that each report corresponds to a single patient but may include multiple ADRs. Initially, 10,252 reports were collected; however, to ensure consistency in the analysis period between finasteride and dutasteride, ADR reports related to finasteride from 2003 and 2004 were excluded, resulting in 10,180 reports. Next, only cases where 5ARIs were the sole suspected drugs, either alone or in combination, were considered. Furthermore, reports involving female patients and individuals under 18 years old were excluded, as these drugs are contraindicated for these populations. After applying these filters, 7777 reports and 38,448 valid ADRs were selected for analysis. During the data analysis, cases with incomplete reports were identified. In these instances, the corresponding variable was classified as “Not specified” or “Unknown.” Additionally, as previously referred, some reports included more than one ADR, and certain ADRs had multiple seriousness criteria. In these cases, the most serious criteria prevailed over the others. In the study of the progression of ADRs considered serious, the same procedure was applied to reports with multiple ADRs and evolutions, always selecting the progression with the highest seriousness criteria. The seriousness criteria were ranked as follows: “Death,” “Life-threatening,” “Hospitalization,” “Disability,” and “Clinically significant condition,” in descending order of seriousness [60].

## 5. Conclusions

The conducted study, involving the 5α-reductase inhibitors finasteride and dutasteride, highlights the importance of pharmacovigilance and its crucial role in assessing the safety profile of medications. Healthcare professionals were the main reporters of the ADRs studied. This study revealed that the age group most affected by ADRs was the “Not Specified” category, followed by individuals aged between 18 and 64 years. Finasteride stood out as the 5ARI most frequently associated with the reported ADRs. Most of the ADRs were not described in the SmPC, with “Cognitive disorder,” “Fatigue,” “Insomnia,” and “Hypertension” being the most notable among them. In contrast, the ADRs described in the SmPC presented the following most reported frequencies: “Erectile dysfunction,” “Decreased libido,” “Depressive symptoms,” “Anxiety,” “Sexual dysfunction,” “Gynecomastia,” and “Testicular pain.” Regarding the ADRs associated with terms from the DME list, “Deafness” was the most frequently reported ADR, followed by “Angioedema,” “Pancreatitis,” “Renal failure,” and “Rhabdomyolysis.” However, it is important to highlight that only “Angioedema” is described in the SmPCs.

Through this study, it became evident that several ADRs associated with 5ARIs highlight the need for more studies to better understand their safety profile. Additionally, persistent and serious ADRs may also represent an important public health and socioeconomic burden due to their potential impact on quality of life, healthcare resource utilization, and work productivity, further reinforcing the importance of continuous post-marketing safety monitoring of these medications.

## Figures and Tables

**Table 1:** Characterization of the population according to age group per drug.

Drugs	Age Group			
	18–64	6585	≥85	Not Specified
Dutasteride	340 (15.93%)	919 (43.04%)	161 (7.54%)	715 (33.49%)
Finasteride	2335 (41.65%	850 (15.16%)	115 (2.05%)	2314 (41.25%)
Dutasteride + Finasteride	9 (32.14%)	6 (21.43%)	1 (3.57%)	12 (42.86%)

**Table 2:** Number of reports of the eight most reported adverse drug reactions described in the Summary of Product Characteristics and their respective frequencies.

ADR	Number of Reports	Frequency Described in the SmPC
Erectile dysfunction	1961	Frequent
Decreased libido	1427	Frequent
Depressive symptoms	1415	Unknown
Anxiety	1007	Unknown
Sexual dysfunction	960	Unknown
Ejaculation disorder	664	Uncommon
Gynecomastia	551	Uncommon
Testicular pain	315	Unknown

ADR: Adverse Drug Reaction; SmPC: Summary of Product Characteristics.

**Table 3:** Number of reports of the four most frequently reported adverse drug reactions not described in the Summary of Product Characteristics (SmPC).

ADR	Number of Reports
Cognitive impairment	623
Fatigue	529
Insomnia	377
Hypertension	276

ADR: Adverse Drug Reaction.

**Table 4:** Relationship between adverse drug reactions from reports with terms belonging to the DME list with the 5α-reductase inhibitor and respective description in the SmPC.

DME Terms	Frequency of ADR Incidence			Description in the SmPC
	Dutasteride	Finasteride	Dutasteride + Finasteride	
Aplastic anemia	2	4	0	Not Described
Angioedema	16	25	0	Described
Blindness	8	1	0	Not Described
Anaphylactic shock	4	3	0	Not Described
Ventricular fibrillation	2	2	0	Not Described
Pulmonary fibrosis	0	3	0	Not Described
Pulmonary arterial hypertension	0	1	0	Not Described
Pulmonary hypertension	0	1	0	Not Described
Bone marrow failure	0	1	0	Not Described
Liver failure	3	6	0	Not Described
Renal failure	7	13	0	Not Described
Drug-induced liver injury	12	5	0	Not Described
Acute kidney injury	1	10	0	Not Described
Progressive multifocal leukoencephalopathy	0	1	0	Not Described
Sudden cardiac death	0	3	0	Not Described
Toxic epidermal necrolysis	1	0	0	Not Described
Liver necrosis	3	6	0	Not Described
Pancytopenia	3	0	0	Not Described
Pancreatitis	10	22	0	Not Described
Bowel perforation	0	1	0	Not Described
Rhabdomyolysis	6	13	0	Not Described
Anaphylactic reaction	1	6	0	Not Described
Stevens–Johnson syndrome	3	1	0	Not Described
Deafness	9	34	0	Not Described
Sensorineural deafness	1	4	0	Not Described
Transmission of an infectious agent via product	1	0	0	Not Described
Immune thrombocytopenia	4	3	0	Not Described

DME: Designated Medical Event; ADR: Adverse Drug Reaction; SmPC: Summary of Product Characteristics.

## Data Availability

The original contributions presented in this study are included in the article. Further inquiries can be directed to the corresponding author.

## Abbreviations

The following abbreviations are used in this manuscript:

ADRs	Adverse Drug Reactions
AGA	Androgenic Alopecia
BPH	Benign Prostatic Hyperplasia
DME	Designated Medical Event
DHT	Dihydrotestosterone
EMA	European Medicines Agency
FDA	Food and Drug Administration
SmPC	Summary of Product Characteristics
5ARIs	5α-Reductase Inhibitors
